# Enhanced Thermoelectric Properties of Oxyselenide Bi_2_O_2_Se via Cl Doping and Microstructure Modulation

**DOI:** 10.3390/ma19081641

**Published:** 2026-04-20

**Authors:** Lele Chen, Ruqing Chen, Yule Huang, Meiqing Liang, Yang Zhou, Danning Ma, Kai Guo

**Affiliations:** 1 School of Physics and Materials Science, Guangzhou University, Guangzhou 510006, China; 32219600076@e.gzhu.edu.cn (L.C.); 32219110046@e.gzhu.edu.cn (R.C.); yulehuang@e.gzhu.edu.cn (Y.H.); lmq051@e.gzhu.edu.cn (M.L.); 32219600080@e.gzhu.edu.cn (Y.Z.); madanning@e.gzhu.edu.cn (D.M.); 2 Key Lab of Si-Based Information Materials & Devices and Integrated Circuits Design, Department of Education of Guangdong Province, Guangzhou 510006, China; 3 Guangdong Provincial Engineering Research Center for Materials Under Extreme Service Environments, Guangzhou University, Guangzhou 510006, China

**Keywords:** Bi_2_O_2_Se, donator doping, microstructure modulation, thermal conductivity, thermoelectric properties

## Abstract

Bi_2_O_2_Se is an emerging oxyselenide semiconductor, noted for its promising thermoelectric properties and excellent chemical stability, and it is often regarded as an n-type counterpart to p-type BiCuSeO. However, its intrinsic thermoelectric figure of merit (*zT*) is severely limited by low electron concentration and high thermal conductivity. In this work, we employed Cl element as donator dopant to substantially enhance the carrier concentration. The room-temperature carrier concentration of Bi_2_O_2_Se_0.98_Cl_0.02_ thereby reached 2.70 × 10^20^ cm^−3^, representing an improvement of two orders of magnitude compared to that of pristine Bi_2_O_2_Se. Subsequently, multiple hot-pressing cycles were applied to the optimized composition Bi_2_O_2_Se_0.98_Cl_0.02_. The process induced significant grain refinement, and the resulting high density of grain boundaries effectively suppressed the lattice thermal conductivity, reducing it to 0.95 W·m^−1^·K^−1^ at 823 K. Eventually, a maximum *zT* of 0.24 was achieved at 823 K for the three-time hot-pressed Bi_2_O_2_Se_0.98_Cl_0.02_ sample, representing a 71% improvement compared with the pristine Bi_2_O_2_Se sample prepared by single hot-pressing (*zT* = 0.14 at 823 K). This work provides a new strategy for enhancing the thermoelectric performance of oxyselenide through the synergistic regulation of doping and microstructure.

## 1. Introduction

The global energy crisis and environmental pollution have become the core challenges to the sustainable development of human society, and in this regard, the development of efficient and clean energy conversion technology has been a research hotspot in the field of materials science and energy [1,2]. Thermoelectric materials can realize the direct mutual conversion of thermal energy and electric energy, with the advantages of non-pollution and noiselessness, which is one of the potential technological paths to solve the problems of energy waste and environmental pollution [3,4].

The thermoelectric energy conversion efficiency of a material is primarily evaluated by the dimensionless figure of merit, *zT*, defined as *zT* = *S*^2^*σT*/*κ*, where *S* is the Seebeck coefficient, *σ* is the electrical conductivity, *T* is the absolute temperature, and *κ* is the total thermal conductivity, comprising both the electronic (*κ*_e_) and lattice (*κ*_L_) contributions [1,5,6]. This expression indicates that a high *zT* requires a material to simultaneously possess a large Seebeck coefficient, high electrical conductivity, and low thermal conductivity. However, achieving a substantial enhancement in *zT* is fundamentally challenging because these three transport parameters are inherently coupled, constituting the key bottleneck for breakthrough thermoelectric performance [5]. For instance, increasing the carrier concentration to enhance electrical conductivity typically reduces the Seebeck coefficient. Furthermore, the electronic thermal conductivity (*κ*_e_) is correlated with electrical conductivity via the Wiedemann–Franz law (*κ*_e_ = *LσT*, where *L* is the Lorenz constant). To decouple these interdependencies and improve *zT*, various optimization strategies have been proposed in recent years. These include reducing materials’ dimensionality [7,8,9], employing point defect engineering [10,11,12,13], developing nanocomposites [14,15], band engineering [16,17,18,19], phonon engineering [20,21], and microstructure modulation [22,23,24,25,26]. The synergistic development and application of these optimization strategies have been pivotal to the advancement of thermoelectric materials.

Currently, state-of-the-art high-*zT* thermoelectric materials primarily include chalcogenides (Bi_2_Te_3_ [27], PbTe [28], GeTe [29], SnSe [30]) Mg_3_(Sb, Bi)_2_ alloys [31], Zintl phases [32], and skutterudites [33]. However, these materials are generally plagued by inherent drawbacks: Te/Ge-based alloys suffer from high cost and elemental scarcity, and lead-containing compounds exhibit severe biotoxicity. In addition, most of these materials display unsatisfactory high-temperature oxidation resistance. These limitations significantly restrict their broad application in mid-to-high temperature ranges and oxygen-containing environments. Consequently, oxide-based thermoelectric materials have emerged as a promising alternative system, offering advantages of excellent high-temperature chemical stability, environmental friendliness, and lower cost. Among various oxide-containing thermoelectrics, the BiCuSeO system stands out as one of the most promising p-type candidates. Yin et al. demonstrated a record-high *zT* of ~1.69 at 767 K for a BiCuSeO-based compound fabricated via an ultrahigh-pressure sintering technique, making it a current research hotspot for p-type oxide-containing thermoelectrics [34]. Nevertheless, a functional thermoelectric device requires the pairing of both p-type and n-type legs to form a closed circuit for energy conversion. In this regard, Pan et al. evaluated the figure of merit of thermoelectric modules based on p-type BiCuSeO and n-type Bi_2_O_2_Se to be approximately 0.8 at 793 K, suggesting that n-type Bi_2_O_2_Se is a viable partner candidate for p-type BiCuSeO-based oxides [35].

**Figure 1:**
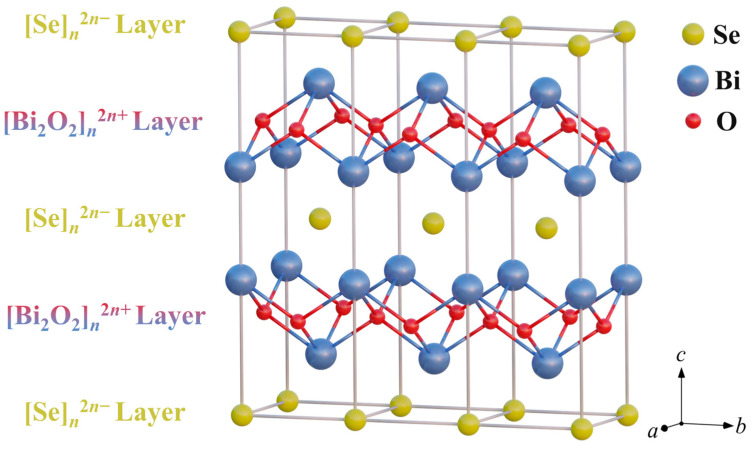
Crystal structure of Bi_2_O_2_Se.

Bi_2_O_2_Se crystallizes in a tetragonal structure with the space group *I*4/mmm (*a* = *b* = 3.891 Å, *c* = 12.21 Å), as illustrated in Figure 1 [36]. It consists of alternating insulating [Bi_2_O_2_]*_n_*^2*n*+^ layers and conducting [Se]*_n_*^2*n*−^ layers stacked along the c-axis [36,37,38]. This distinctive layered structure endows the material with remarkable physicochemical properties. From a thermoelectric perspective, Bi_2_O_2_Se exhibits intrinsically low lattice thermal conductivity, a high Seebeck coefficient, and high charge carrier mobility. However, its relatively low intrinsic carrier concentration results in insufficient electrical conductivity, which limits the overall thermoelectric performance [38,39]. Since the initial report on the thermoelectric properties of Bi_2_O_2_Se by Ruleova et al. in 2010 [40], subsequent research has primarily focused on performance optimization through elemental doping to introduce additional charge carriers or modify the electronic band structure. Most studies involve doping at the Bi site with elements such as Sn [41], Ge [42,43], La [44], Ta [45], Ce [46], Nb [47,48], Sb [49], and Ti [50]. Reports also exist on Se site doping with Cl [51] and Te [52], and O site doping with Te [53] and S [54]. In addition to doping, strategies such as texturing via shear exfoliation [35,50,55] and forming composites with other materials are also employed to enhance material performance [56,57].

To address the issues of low carrier concentration and high in-plane lattice thermal conductivity induced by its layered structure, which severely limit the thermoelectric performance of pristine Bi_2_O_2_Se, this work aims to develop a synergistic regulation strategy of donor doping and microstructure engineering. Specifically, Cl is selected to dope Se sites for optimizing carrier transport properties, and multiple hot-pressing cycles are adopted to tailor grain microstructure and suppress phonon transport. This study is expected to realize the decoupling of electron–phonon transport and provide a feasible approach for improving the thermoelectric properties of oxyselenide materials.

## 2. Materials and Methods

**Powder Synthesis**. Bi_2_O_2_Se_1−*x*_Cl*_x_* (*x* = 0, 0.02, 0.04) powder was synthesized via a solid-state reaction [40,48]. The detailed procedure is as follows. First, high-purity elemental powders of Bi (99.99%, Aladdin, Shanghai, China), Se (99.99%, Aladdin), Bi_2_O_3_ (99.99%, Aladdin), and BiCl_3_ (99.99%, Aladdin) were precisely weighed according to the stoichiometric ratio inside an argon-filled glovebox (Universal, Mikrouna, Shanghai, China). The mixed powder was then loaded into a dual-can high-energy ball mill (SGQM-III, Shanghai Songyao Materials Equipment Co., Ltd., Shanghai, China) and milled at 600 rpm for 2 h with a ball-to-powder weight ratio of 5:1. The ball-milling process was designed to achieve uniform mixing of the initial raw materials. Subsequently, the as-milled powder mixture was pressed into blocks with a diameter of 12.7 mm. These blocks were sealed in an evacuated quartz tube using a vacuum sealing system (FYFK2, Changzhou Daye Energy Technology Co., Ltd., Changzhou, China). The sealed tube was placed in a muffle furnace (VBF-1200X, Hefei Kejing Materials Technology Co., Ltd., Hefei, China) and subjected to the following heating profile: heating to 573 K at a rate of 5 K·min^−1^ and holding for 6 h, followed by heating to 773 K at a rate of 1 K·min^−1^ and holding for 24 h. Finally, the furnace was cooled to room temperature naturally. The resulting product was lightly ground for 1–2 min to obtain the final Bi_2_O_2_Se_1−*x*_Cl*_x_* powder. The manual grinding step was performed to guarantee the compositional uniformity of the sample during the subsequent hot-pressing process.

**Hot-Pressing Sintering**. The as-synthesized Bi_2_O_2_Se_1−*x*_Cl*_x_* powder was consolidated via a vacuum hot-press sintering furnace (OTF-1200X-VHP4, Hefei Kejing Materials Technology Co., Ltd, Hefei, China) [48]. The powder was first loaded into a cylindrical graphite die with an inner diameter of 11 mm and sintered at 923 K under 50 MPa for 1 h. Subsequently, the obtained blocks were transferred into larger graphite dies (inner diameters of 12 mm and 12.7 mm) for the second and third rounds of hot-pressing, respectively, using the same temperature, pressure, and dwelling time as the first cycle. All hot-pressing procedures were carried out under vacuum.

**Performance Characterization**. Phase analysis was performed using X-ray diffraction (XRD, D8 ADVANCE, Bruker Corporation, Billerica, MA, USA) with Cu-Kα radiation (λ = 1.5406 Å). Data were collected in a 2*θ* range from 10° to 80° with a step size of 0.02° and a scanning rate of 2° min^−1^, for the powder sample. The surface morphology of the samples was examined by scanning electron microscopy (SEM, JSM-IT800, JEOL Ltd., Tokyo, Japan) operated at an accelerating voltage of 10 kV and a working distance of 10 mm in high-vacuum mode, using the secondary electron detector (SED). Elemental distribution was analyzed via energy-dispersive X-ray spectroscopy (EDS) mapping attached to the SEM. Furthermore, grain size analysis was performed using the electron backscatter diffraction (EBSD) accessory equipped with the same SEM system. EBSD patterns were acquired from the sample surface, and the corresponding grain size distribution was determined using the instrument’s dedicated analysis software (AZtecCrystal2.1). The electrical conductivity (*σ*) and Seebeck coefficient (*S*) were measured simultaneously from 303 K to 823 K in a helium atmosphere using a commercial thermoelectric property measurement system (CTA-3S, Beijing Cryoall Science and Technology Co., Ltd., Beijing, China). Room-temperature carrier concentration (*n*) and carrier mobility (*μ*) were determined by Hall effect measurements using the van der Pauw method under a magnetic field of 1.4 T. The thermal conductivity (*κ*) was calculated using the formula *κ* = *DρC_p_*, where the thermal diffusivity (*D*) was measured from 303 K to 823 K in an argon atmosphere using a laser flash analyzer (LFA-457, Netzsch, Selb, Germany). The density (*ρ*) was determined by the Archimedes method, with all synthesized samples achieving a relative density above 90% (Table S1, where the theoretical density of Bi_2_O_2_Se is 9.50 g·cm^−3^). The specific heat capacity (*C_p_*) was estimated using the Dulong–Petit law, *C_p_* = 3*n*R/*M*, where R is the ideal gas constant, *n* is the number of atoms per formula unit, and *M* is the molar mass. The thermoelectric figure of merit, *zT*, was then calculated as *zT* = *S*^2^*σ*/*κ*. The measurement uncertainties for electrical conductivity (*σ*), Seebeck coefficient (*S*) and thermal conductivity (*κ*) were determined to be approximately ±3%, ±5% and ±5%, respectively. The overall relative uncertainty for the calculated *zT* values was estimated to be about 10–15%.

## 3. Results and Discussion

**Figure 2:**
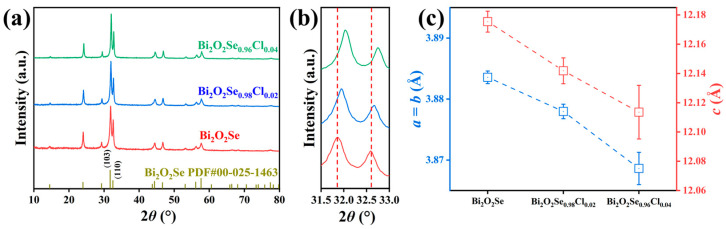
(**a**) Room-temperature powder X-ray diffraction patterns of Bi_2_O_2_Se_1−*x*_Cl*_x_* (*x* = 0, 0.02, 0.04). (**b**) Magnified diffraction peaks between 31.5° and 33°. (**c**) Lattice constants of Bi_2_O_2_Se_1−*x*_Cl*_x_* (*x* = 0, 0.02, 0.04).

Figure 2a shows the room-temperature X-ray diffraction patterns of as-synthesized Bi_2_O_2_Se_1−*x*_Cl*_x_* (*x* = 0, 0.02, 0.04). All observed diffraction peaks for each sample can be precisely indexed to the standard pattern of Bi_2_O_2_Se, with no detectable impurity peaks. This indicates the successful synthesis of high-purity Bi_2_O_2_Se-based materials.

**Figure 3:**
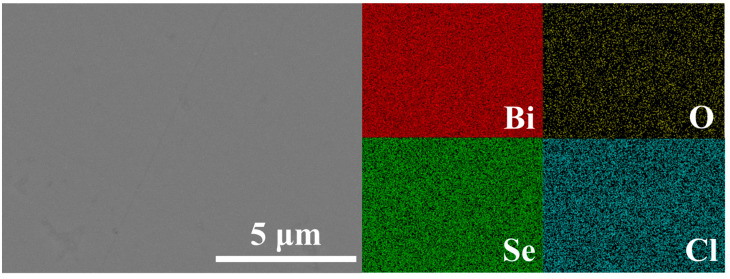
The SEM image of sample Bi_2_O_2_Se_0.98_Cl_0.02_ and corresponding EDS elemental mapping.

Notably, as shown in Figure 2b, after Cl doping, the (103) and (110) diffraction peaks exhibit a distinct shift toward higher angles, suggesting a possible contraction of the crystal lattice induced by Cl incorporation. Accordingly, the lattice parameters were calculated from the XRD data, with the results presented in Figure 2c. Indeed, Cl doping leads to a contraction of the lattice, which is particularly pronounced along the *c*-axis. This anisotropic contraction can be attributed to the substitutional doping of Cl^−^ for Se^2−^. Since the ionic radius of Cl^−^ (~1.81 Å) is smaller than that of Se^2−^ (~1.96 Å), and the doping site is located within the interlayer region (Figure 1), the compression effect is more significant along the *c*-axis. The SEM image and corresponding EDS elemental mapping for the Bi_2_O_2_Se_0.98_Cl_0.02_ sample are presented in Figure 3. The SEM micrograph reveals a relatively dense and smooth surface morphology, with the visible scratches attributed to the polishing process. EDS elemental mapping demonstrates a homogeneous distribution of the constituent elements Bi, O, Se, and Cl. This uniform elemental distribution, combined with the XRD observations of lattice contraction, provides corroborating evidence that Cl has been successfully incorporated into the Bi_2_O_2_Se crystal lattice via substitutional doping, rather than forming secondary phases.

Bi_2_O_2_Se crystallizes in a layered structure, which is expected to result in anisotropic charge and/or heat transport. Consequently, a complete characterization calls for measurements on bulk samples along various directions. Figure S1 displays the electrical and thermal transport properties of the Bi_2_O_2_Se_0.98_Cl_0.02_ sample measured perpendicular to the pressing direction (Bi_2_O_2_Se_0.98_Cl_0.02_ ⊥) and parallel to the pressing direction (Bi_2_O_2_Se_0.98_Cl_0.02_ ∥). The Seebeck coefficients along both directions are nearly identical across the entire measured temperature range, and the thermal conductivities are also very similar. Benefiting from the materials’ layered nature, the electrical conductivity measured perpendicular to the pressing direction is consistently higher than that measured parallel to the pressing direction. Consequently, both the power factor and the thermoelectric figure of merit (*zT*) are superior along the perpendicular direction. This anisotropic behavior aligns with the findings of Li et al., who likewise reported slightly higher power factors and *zT* values in the direction perpendicular to the pressing axis in Bi_2_O_2_Se [48]. For this reason, all electrical and thermal transport properties reported in this work were measured along the direction perpendicular to the pressing axis.

**Figure 4:**
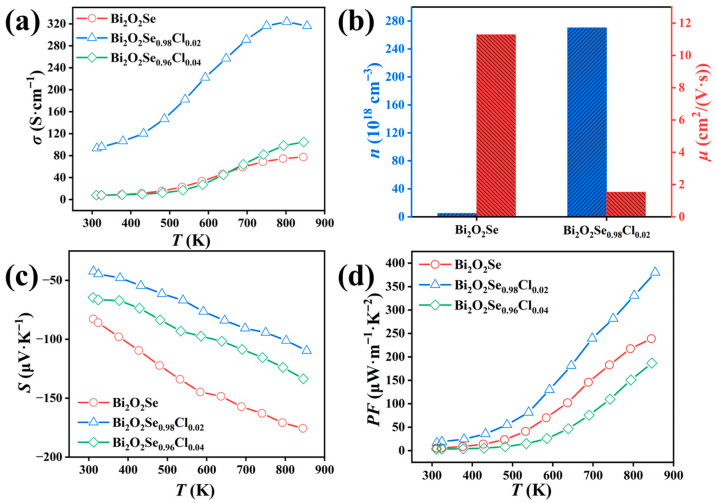
(**a**) Temperature-dependent electrical conductivity, (**b**) room-temperature carrier concentration and mobility, (**c**) temperature-dependent Seebeck coefficient, and (**d**) temperature-dependent power factor of Bi_2_O_2_Se_1−*x*_Cl*_x_* (*x* = 0, 0.02, 0.04).

To elucidate the effect of Cl doping on the electrical transport properties of Bi_2_O_2_Se, the key electrical transport parameters were measured. As shown in Figure 4a, the electrical conductivity (*σ*) of all samples increases with temperature, a characteristic behavior of a non-degenerate semiconductor. Upon Cl doping, the room-temperature *σ* is dramatically enhanced from 7.72 S·cm^−1^ for pristine Bi_2_O_2_Se to 93.53 S·cm^−1^ for the Bi_2_O_2_Se_0.98_Cl_0.02_ sample. The *σ* of the Cl^−^-doped sample remains significantly higher across the entire measured temperature range. This substantial improvement originates primarily from a remarkable increase in the room-temperature carrier concentration (*n*) from 4.93 × 10^18^ cm^−3^ to 2.70 × 10^20^ cm^−3^, as shown in Figure 4b. Notably, the enhanced room-temperature carrier concentration in this work is higher than those reported in previous studies with doping at the Se site of Bi_2_O_2_Se [51,52]. As the absolute Seebeck coefficient (|*S*|) is inversely proportional to the carrier concentration, the |*S*| of the Bi_2_O_2_Se_0.98_Cl_0.02_ sample exhibits a marked reduction compared to the pristine Bi_2_O_2_Se, decreasing from 82.77 μV·K^−1^ to 42.34 μV·K^−1^ at room temperature, as shown in Figure 4c. This observation is consistent with its significantly increased carrier density. Furthermore, the negative sign of the Seebeck coefficient for all samples confirms their n-type conduction behavior. Consequently, the power factor (*PF* = *S*^2^*σ*) was calculated and is plotted in Figure 4d. Benefiting from the order-of-magnitude enhancement in *σ* and a comparably moderate decrease in |*S*|, the Bi_2_O_2_Se_0.98_Cl_0.02_ sample achieves a markedly improved *PF*. It exhibits the highest *PF* value across the entire temperature range measured. These results collectively demonstrate that Cl doping effectively optimizes the carrier concentration and significantly enhances the electrical transport performance of Bi_2_O_2_Se.

Notably, although the room-temperature carrier concentration is increased by two orders of magnitude via Cl donor doping, the enhancement of electrical conductivity and the reduction in Seebeck coefficient are relatively moderate. This phenomenon can be systematically explained by the following physical mechanisms. First, high-concentration Cl doping introduces strong ionized impurity scattering, which reduces the carrier mobility (Figure 4b) and offsets the positive contribution of increased carrier concentration to electrical conductivity. Second, according to the single-parabolic-band model, the Seebeck coefficient follows the relationship |*S*| ∝ m*n^−2/3^, where m* is the carrier effective mass and *n* is the carrier concentration. This indicates that the Seebeck coefficient is not only inversely proportional to the two-thirds power of carrier concentration, but also proportional to the carrier effective mass. As shown in the room-temperature Pisarenko plot (Figure S2), the pristine Bi_2_O_2_Se sample well follows the theoretical curve with m* = 0.12*m*_e_, where the *m*_e_ is the free electron mass, while the Bi_2_O_2_Se_0.98_Cl_0.02_ sample lies above the curve with a calculated effective mass of 0.87*m*_e_, indicating a significantly increased carrier effective mass after Cl doping. The increased m* can partially counteract the decrease in |*S*| caused by rising carrier concentration, thus leading to a moderate reduction in |*S*|. In addition, Hall measurements are only performed at room temperature, which cannot reflect the temperature-dependent evolution of carrier transport at high temperatures up to 823 K.

**Figure 5:**
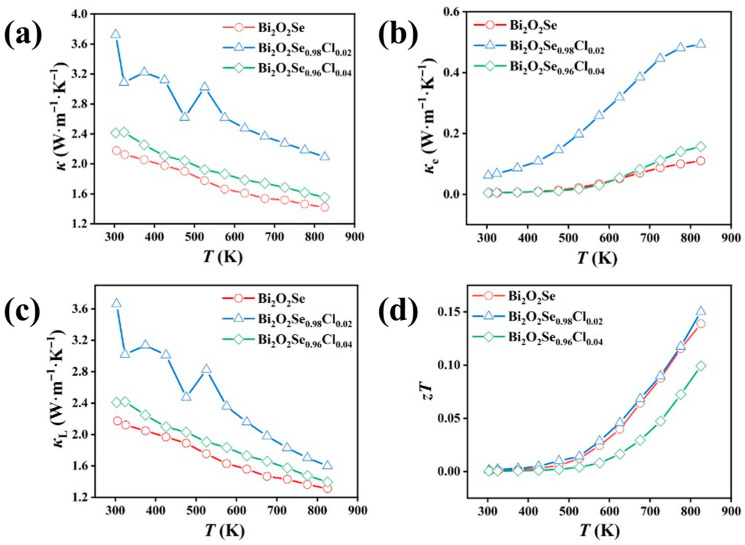
(**a**) Temperature-dependent total thermal conductivity, (**b**) temperature-dependent electronic thermal conductivity, (**c**) temperature-dependent lattice thermal conductivity, and (**d**) temperature-dependent figure of merit *zT* of Bi_2_O_2_Se_1−*x*_Cl*_x_* (*x* = 0, 0.02, 0.04).

Figure 5a presents the total thermal conductivity (*κ*) of the samples Bi_2_O_2_Se_1−_*_x_*Cl*_x_* (*x* = 0, 0.02, 0.04). The *κ* for all samples decreases with increasing temperature, indicating that the lattice thermal conductivity (*κ*_L_) is the dominant contributor to the *κ*, while the electronic component (*κ*_e_) is minimal. This is further corroborated by the calculated electronic thermal conductivity *κ*_e_ (Figure 5b) and lattice thermal conductivity *κ*_L_ (Figure 5c). *κ*_e_ and *κ*_L_ were calculated using the following formulas:

*κ*_e_ = *LσT* (1)

*κ*_L_ = *κ* − *κ*_e_ (2)

*L* = 1.5 + e^−|*S*|/116^ (3)

Here, *L* is the Lorentz constant (Figure S3) and *S* is the Seebeck coefficient. The intrinsically low lattice thermal conductivity of pristine Bi_2_O_2_Se is primarily attributed to the layered structure-induced anharmonicity and strong phonon scattering caused by its abundant native Se vacancies. Typically, point defects introduced by doping are expected to further enhance phonon scattering and reduce lattice thermal conductivity. However, in this work, low-concentration Cl doping resulted in a slight increase in *κ*_L_, from 1.31 W·m^−1^·K^−1^ for pristine Bi_2_O_2_Se to 1.60 W·m^−1^·K^−1^ for the Bi_2_O_2_Se_0.98_Cl_0.02_ sample at 823 K. The reason is due to the high density of the sample (Table S1). Despite the overall increase in *κ* for the Bi_2_O_2_Se_0.98_Cl_0.02_ sample, which is attributed to its significantly enhanced *κ*_L_, the calculated thermoelectric figure of merit, *zT* (Figure 5d), surpasses that of the pristine sample across the entire measured temperature range. This improvement is primarily attributed to the dramatic enhancement in the power factor. Specifically, the Bi_2_O_2_Se_0.98_Cl_0.02_ sample achieves a peak *zT* of approximately 0.15 at 823 K.

Despite the improved electrical transport from Cl doping, the thermal conductivity (*κ*), particularly its lattice part (*κ*_L_), can be further lowered—largely due to persistent phonon scattering limitations. To this end, the effect of a multiple-cycle hot-pressing process on the microstructure and thermoelectric performance of the optimal composition was investigated. Based on the previously identified best-performing composition (Bi_2_O_2_Se_0.98_Cl_0.02_), samples were subjected to one, two, and three cycles of hot-pressing, designated as Bi_2_O_2_Se_0.98_Cl_0.02_-1, Bi_2_O_2_Se_0.98_Cl_0.02_-2 and Bi_2_O_2_Se_0.98_Cl_0.02_-3, respectively.

**Figure 6:**
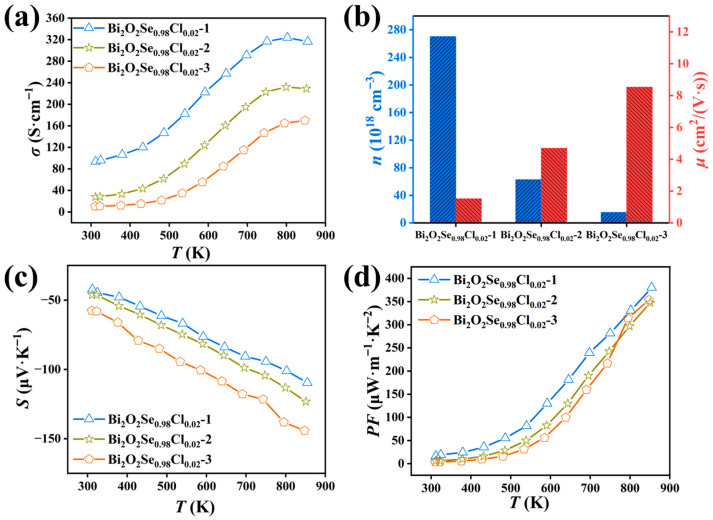
(**a**) Temperature-dependent electrical conductivity, (**b**) room-temperature carrier concentration and mobility, (**c**) temperature-dependent Seebeck coefficient, and (**d**) temperature-dependent power factor of Bi_2_O_2_Se_0.98_Cl_0.02_ under different hot-pressing cycles.

**Figure 7:**
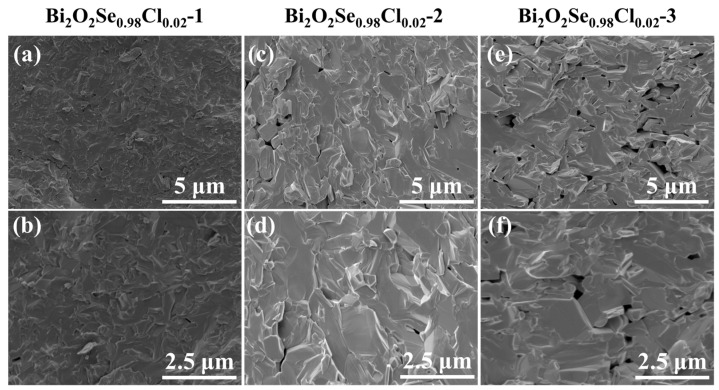
Fracture surface SEM images of the hot-pressed Bi_2_O_2_Se_0.98_Cl_0.02_ samples: (**a**,**b**) Bi_2_O_2_Se_0.98_Cl_0.02_-1; (**c**,**d**) Bi_2_O_2_Se_0.98_Cl_0.02_-2; and (**e**,**f**) Bi_2_O_2_Se_0.98_Cl_0.02_-3.

As shown in Figure 6a, the electrical conductivity (*σ*) decreases progressively with an increasing number of hot-pressing cycles across the entire temperature range. Hall effect measurements (Figure 6b) reveal that this phenomenon is primarily attributed to a significant reduction in carrier concentration, while the carrier mobility remains largely unchanged. Considering the hot-pressing temperature (923 K) and the volatile nature of Cl, it is speculated that during the repeated hot-pressing cycles, a portion of the Cl atoms substituted at the Se sites may have been lost via volatilization. This loss would reduce the effective donor concentration, consequently leading to the observed decrease in carrier concentration. Notably, the cross-sectional SEM characterization results (Figure 7) show a clear increase in material porosity with the number of hot-pressing cycles. This microstructural evolution provides indirect support for the hypothesis of Cl volatilization, and the increased porosity also accounts for the decrease in sample density (Table S1). In addition, XRD measurements performed perpendicular to the pressing direction (Figure S4) show that the lattice constants gradually increase with the number of hot-pressing cycles, which provides strong evidence for the volatilization of Cl. It is important to emphasize that even after three hot-pressing cycles, the carrier concentration of Bi_2_O_2_Se_0.98_Cl_0.02_-3 remains substantially higher than that of pristine Bi_2_O_2_Se. Furthermore, as shown in Figure 6c, the absolute Seebeck coefficient (|*S*|) increases with the number of hot-pressing cycles across the entire measured temperature range, a trend also primarily driven by the decreased carrier concentration. Despite the modest increase in |*S*|, its positive contribution to the power factor (*PF* = *S*^2^*σ*) cannot compensate for the significant degradation in *σ* caused by repeated hot-pressing. Consequently, as shown in Figure 6d, the *PF* values for the Bi_2_O_2_Se_0.98_Cl_0.02_-2 and Bi_2_O_2_Se_0.98_Cl_0.02_-3 samples are lower than that of the Bi_2_O_2_Se_0.98_Cl_0.02_-1 sample. This indicates that, for the Bi_2_O_2_Se_0.98_Cl_0.02_ composition, a single hot-pressing cycle already yields electrical transport properties at or near their optimum state.

**Figure 8:**
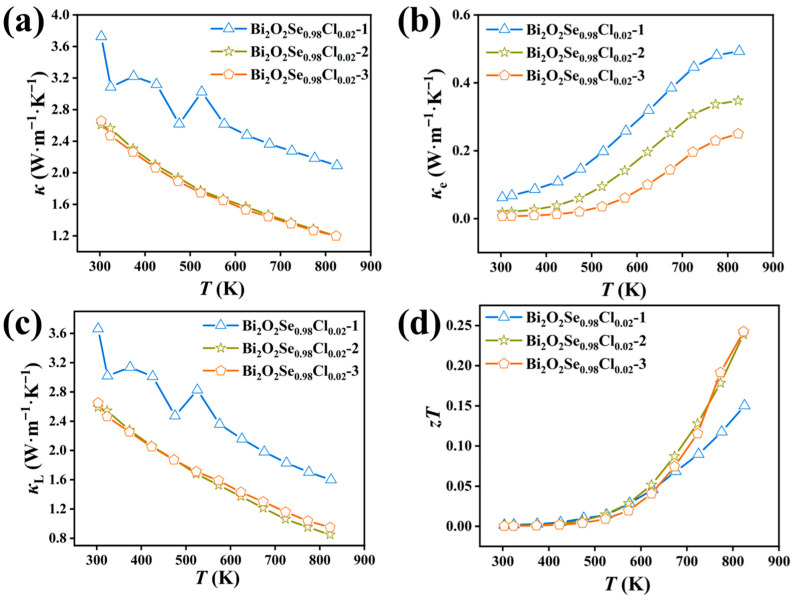
(**a**) Temperature-dependent total thermal conductivity, (**b**) temperature-dependent electronic thermal conductivity, (**c**) temperature-dependent lattice thermal conductivity, and (**d**) temperature-dependent figure of merit *zT* of Bi_2_O_2_Se_0.98_Cl_0.02_ under different hot-pressing cycles.

**Figure 9:**
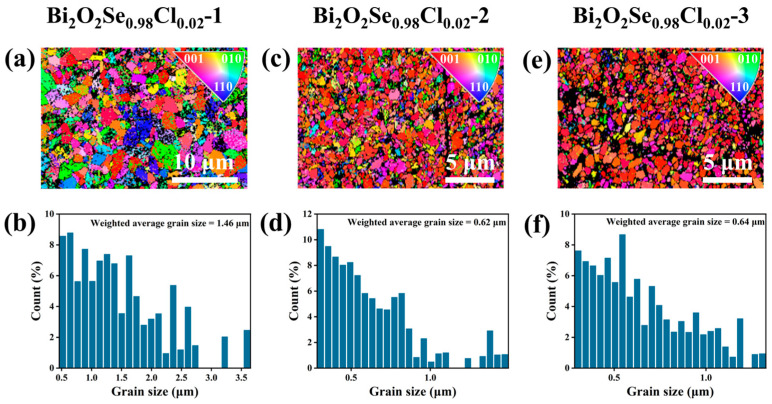
Electron backscatter diffraction (EBSD) maps and the corresponding grain size distribution histograms of the samples: (**a**,**b**) Bi_2_O_2_Se_0.98_Cl_0.02_-1; (**c**,**d**) Bi_2_O_2_Se_0.98_Cl_0.02_-2; and (**e**,**f**) Bi_2_O_2_Se_0.98_Cl_0.02_-3.

**Figure 10:**
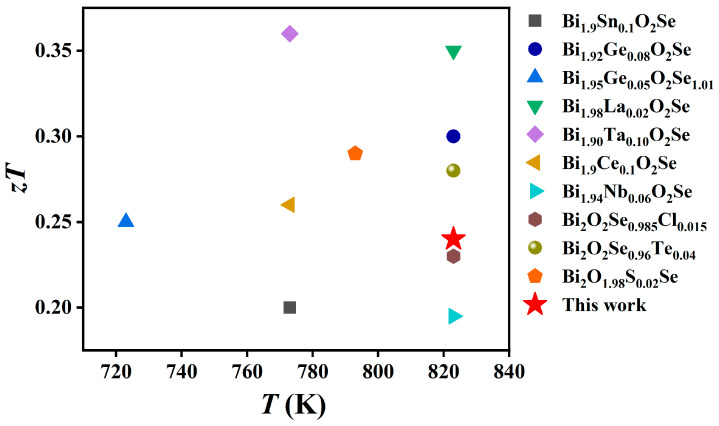
Comparison of the maximum thermoelectric figure of merit (*zT*) values for Bi_2_O_2_Se-based materials prepared by conventional solid-state reaction, spark plasma sintering (SPS) or vacuum hot-pressing sintering, with the red star representing the result of this work [41,42,43,44,45,46,48,51,52,54].

Having established the effect of multiple hot-pressing cycles on the electrical transport properties, we further analyzed the evolution of the thermal transport properties to elucidate how the hot-pressing process synergistically enhances the thermoelectric figure of merit (*zT*) through microstructural engineering. As shown in Figure 8a, the total thermal conductivity (*κ*) exhibits a progressive decrease with an increasing number of hot-pressing cycles. At 823 K, the Bi_2_O_2_Se_0.98_Cl_0.02_-3 sample shows a reduction in *κ* of approximately 43% compared to the Bi_2_O_2_Se_0.98_Cl_0.02_-1 sample, indicating a substantial optimization. To identify the origin of this reduction, the electronic (*κ*_e_) and lattice (*κ*_L_) thermal conductivities were calculated and are presented in Figure 8b and Figure 8c, respectively. The *κ*_L_ remains the dominant contributor to the *κ* in all samples. Consequently, the observed decrease in *κ* is primarily governed by the reduction in *κ*_L_. Compared to the Bi_2_O_2_Se_0.98_Cl_0.02_-1 sample, the Bi_2_O_2_Se_0.98_Cl_0.02_-2 and Bi_2_O_2_Se_0.98_Cl_0.02_-3 samples exhibit a significantly lower *κ*_L_. The *κ*_L_ of the Bi_2_O_2_Se_0.98_Cl_0.02_-3 sample is reduced to 0.95 W·m^−1^·K^−1^ at 823 K, which is comparable to those reported in previous studies [44,48]. This is primarily attributed to the repeated plastic deformation and recrystallization during the multiple hot-pressing cycles, which lead to further grain refinement. As evidenced by the data in Figure 9, the grain size substantially decreases after the second and third hot-pressing steps. This results in a marked increase in grain boundary density. These grain boundaries act as effective phonon scattering centers, strongly scattering phonons and thereby significantly suppressing *κ*_L_. In addition, the increased porosity induced by multiple hot-pressing leads to reduced sample density, which further results in the decrease in *κ*_L_. By integrating the electrical and thermal transport properties, the *zT* values were calculated, as plotted in Figure 8d. Although the electrical performance is somewhat compromised by multiple hot-pressing, the significantly greater reduction in *κ*_L_ results in a net enhancement of *zT*. Consequently, the *zT* of the Bi_2_O_2_Se_0.98_Cl_0.02_-3 sample surpasses that of the Bi_2_O_2_Se_0.98_Cl_0.02_-1 sample, reaching a peak value of ~0.24 at 823 K. This represents a noteworthy enhancement of approximately 71% compared to the *zT* value of 0.14 for the undoped, once-hot-pressed Bi_2_O_2_Se sample. To evaluate the thermoelectric performance of this work, a comparison with previously reported Bi_2_O_2_Se-based materials prepared by conventional solid-state reaction, spark plasma sintering (SPS) or vacuum hot-pressing sintering is presented in Figure 10. Due to significant differences in preparation processes, this comparison excludes samples fabricated by liquid-phase shear exfoliation. The maximum *zT* value of 0.24 obtained in this work is comparable to those of most cation/anion-doped Bi_2_O_2_Se bulks with the same sintering process, demonstrating that Cl doping combined with repeated hot-pressing is an effective way to optimize the thermoelectric properties of Bi_2_O_2_Se.

## 4. Conclusions

This study demonstrates the synergistic enhancement of the thermoelectric performance of n-type Bi_2_O_2_Se through a combined strategy of Cl doping at the Se site and a multiple-cycle hot-pressing process. The results indicate that Cl acts as an effective donor dopant, increasing the room-temperature carrier concentration of Bi_2_O_2_Se by two orders of magnitude, which leads to a remarkable improvement in electrical conductivity and power factor. Subsequently, applying multiple hot-pressing cycles to the optimal composition, Bi_2_O_2_Se_0.98_Cl_0.02_, led to significant grain refinement and an increased density of grain boundaries. These grain boundaries serve as highly effective phonon scattering centers, reducing the lattice thermal conductivity by approximately 41% at 823 K compared to the once-hot-pressed (Bi_2_O_2_Se_0.98_Cl_0.02_-1) sample. Consequently, the Bi_2_O_2_Se_0.98_Cl_0.02_ sample subjected to three hot-pressing cycles achieved a *zT* value of ~0.24 at 823 K. This represents a 71% enhancement compared with the undoped Bi_2_O_2_Se sample fabricated via single hot-pressing (*zT* = 0.14 at 823 K), as the significant suppression of thermal transport outweighs the modest degradation in electrical properties. This work not only confirms the feasibility of decoupling electron and phonon transport through combined doping and microstructure engineering but also provides an effective and scalable strategy for developing high-performance oxyselenide thermoelectric materials. To address the issues of Cl dopant volatilization and reduced effective doping concentration during multiple hot-pressing, it is theoretically feasible to compensate for the doping loss by increasing the initial Cl doping content or adopting dopants with low volatility, which is expected to achieve higher thermoelectric performance in Bi_2_O_2_Se-based materials.

## Data Availability

The original contributions presented in this study are included in the article/Supplementary Materials. Further inquiries can be directed at the corresponding author.

## Supplementary Materials

The following supporting information can be downloaded at: https://www.mdpi.com/article/10.3390/ma19081641/s1, Figure S1: (a) The temperature dependence of electrical conductivity, (b) the temperature dependence of Seebeck coefficient, (c) the temperature dependence of power factor, (d) the temperature dependence of total thermal conductivity, and (e) the temperature dependence of the figure of merit *zT* for the sample Bi_2_O_2_Se_0.98_Cl_0.02_ measured along various directions; Figure S2: Room-temperature |*S*| as a function of carrier concentration. The solid line is a Pisarenko plot at 303 K; Figure S3: (a) Temperature dependence of the Lorentz constant for Bi_2_O_2_Se_1−*x*_Cl*_x_* (*x* = 0, 0.02, 0.04). (b) Temperature dependence of the Lorentz constant for Bi_2_O_2_Se_0.98_Cl_0.02_-1, Bi_2_O_2_Se_0.98_Cl_0.02_-2, and Bi_2_O_2_Se_0.98_Cl_0.02_-3; Figure S4: (a) Room temperature X-ray diffraction patterns of Bi_2_O_2_Se_0.98_Cl_0.02_-1, Bi_2_O_2_Se_0.98_Cl_0.02_-2 and Bi_2_O_2_Se_0.98_Cl_0.02_-3. (b) Magnified diffraction peaks between 31.5° and 33°. (c) Lattice constants of Bi_2_O_2_Se_0.98_Cl_0.02_-1, Bi_2_O_2_Se_0.98_Cl_0.02_-2 and Bi_2_O_2_Se_0.98_Cl_0.02_-3. Table S1: The density of samples Bi_2_O_2_Se_1−*x*_Cl*_x_* (*x* = 0, 0.02, 0.04), Bi_2_O_2_Se_0.98_Cl_0.02_-2, and Bi_2_O_2_Se_0.98_Cl_0.02_-3.
