# Supplementary material for: Enhanced Thermoelectric Properties of Oxyselenide Bi2O2Se via Cl Doping and Microstructure Modulation
Source: Materials (Basel). 2026 Apr 20;19(8):1641. doi: 10.3390/ma19081641 (PMC13118045; doi:10.3390/ma19081641)
Supplement: Supplementary file 1 [file materials-19-01641-s001.zip › materials-4259694-supplementary.pdf]

Enhanced Thermoelectric Properties of Oxyselenide Bi_2O_2Se via Cl Doping and Microstructure Modulation

Lele Chen [1], Ruqing Chen [1], Yule Huang [1], Meiqing Liang [1], Yang Zhou [1], Danning Ma [1] and Kai Guo [1,2,3,*]

1 School of Physics and Materials Science, Guangzhou University, Guangzhou 510006, China; 32219600076@e.gzhu.edu.cn (L.C.); 32219110046@e.gzhu.edu.cn (R.C.); yulehuang@e.gzhu.edu.cn (Y.H.); lmq051@e.gzhu.edu.cn (M.L.); 32219600080@e.gzhu.edu.cn (Y.Z.); madanning@e.gzhu.edu.cn (D.M.)

2 Key Lab of Si-Based Information Materials & Devices and Integrated Circuits Design, Department of Education of Guangdong Province, Guangzhou 510006, China

3 Guangdong Provincial Engineering Research Center for Materials under Extreme Service Environments, Guangzhou University, Guangzhou 510006, China

* Correspondence: kai.guo@gzhu.edu.cn

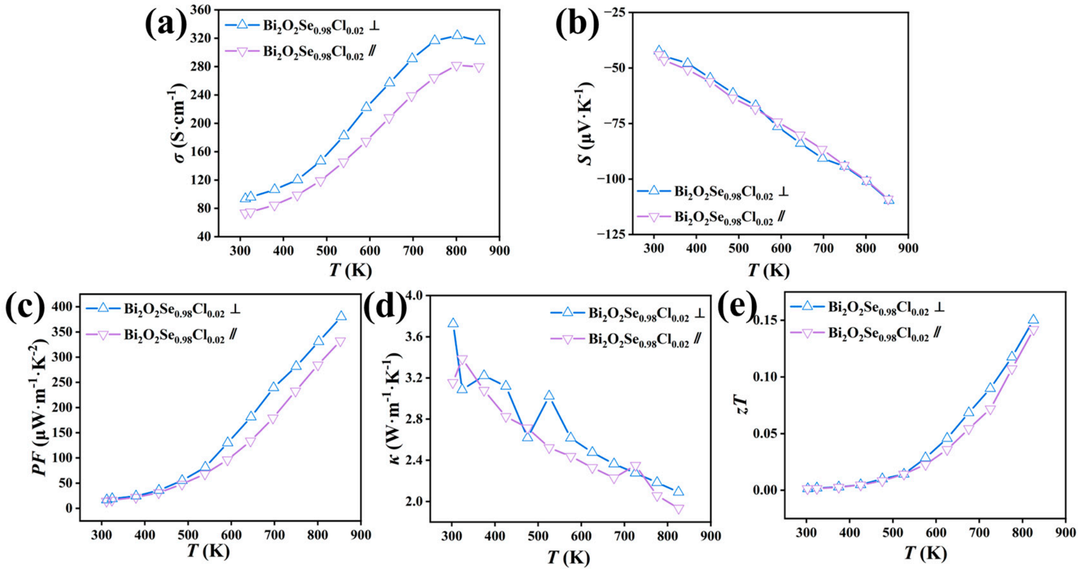

Figure S1. (**a**) The temperature dependence of electrical conductivity, (**b**) the temperature dependence of Seebeck coefficient, (**c**) the temperature dependence of power factor, (**d**) the temperature dependence of total thermal conductivity, and (**e**) the temperature dependence of the figure of merit zT for the sample $Bi_2O_2Se_{0.98}Cl_{0.02}$ measured along various directions.

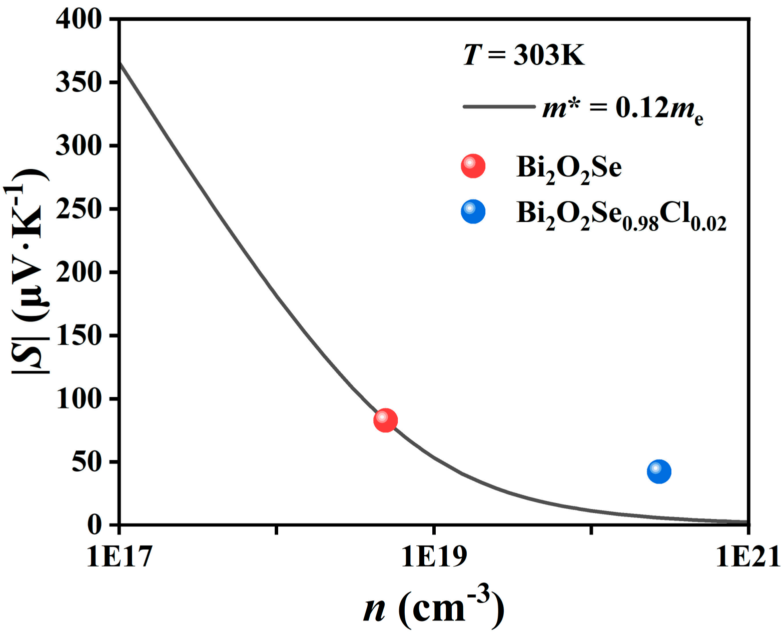

Figure S2. Room-temperature |S| as a function of carrier concentration. The solid line is a Pisarenko plot at 303 K.

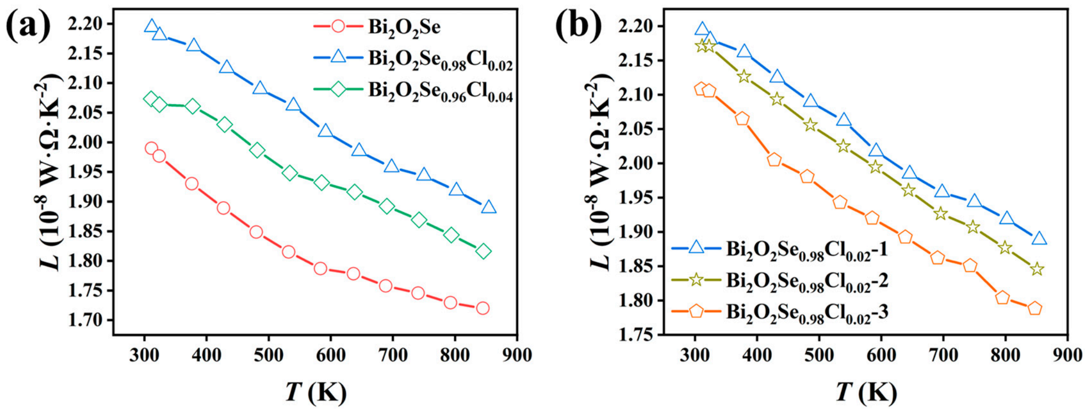

Figure S3. (**a**) Temperature dependence of the Lorentz constant for $Bi_2O_2Se_{1-x}Cl_x$ (x = 0, 0.02, 0.04). (**b**) Temperature dependence of the Lorentz constant for $Bi_2O_2Se_{0.98}Cl_{0.02}$-1, $Bi_2O_2Se_{0.98}Cl_{0.02}$-2, and $Bi_2O_2Se_{0.98}Cl_{0.02}$-3.

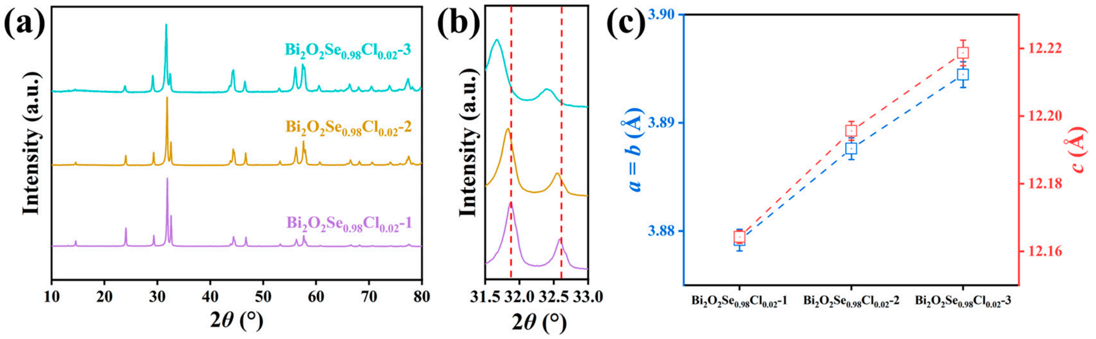

Figure S4. (**a**) Room temperature X-ray diffraction patterns of $Bi_2O_2Se_{0.98}Cl_{0.02}$-1, $Bi_2O_2Se_{0.98}Cl_{0.02}$-2 and $Bi_2O_2Se_{0.98}Cl_{0.02}$-3. (**b**) Magnified diffraction peaks between 31.5° and 33°. (**c**) Lattice constants of $Bi_2O_2Se_{0.98}Cl_{0.02}$-1, $Bi_2O_2Se_{0.98}Cl_{0.02}$-2 and $Bi_2O_2Se_{0.98}Cl_{0.02}$-3.

Table S1. The density of samples $Bi_2O_2Se_{1-x}Cl_x$ (x = 0, 0.02, 0.04), $Bi_2O_2Se_{0.98}Cl_{0.02}$-2, and $Bi_2O_2Se_{0.98}Cl_{0.02}$-3.

**Compositions**	**Density (g·cm^{-3})**
Bi_2O_2Se	9.1019
$Bi_2O_2Se_{0.98}Cl_{0.02}$	9.4260
$Bi_2O_2Se_{0.96}Cl_{0.04}$	8.8037
$Bi_2O_2Se_{0.98}Cl_{0.02}$-2	9.1813
$Bi_2O_2Se_{0.98}Cl_{0.02}$-3	8.9096
